# Cisplatin selects short forms of the mitochondrial DNA OriB variant (16184–16193 poly-cytosine tract), which confer resistance to cisplatin

**DOI:** 10.1038/srep46240

**Published:** 2017-04-10

**Authors:** Taku Amo, Naomi Kamimura, Hiromasa Asano, Sadamitsu Asoh, Shigeo Ohta

**Affiliations:** 1 Department of Biochemistry and Cell Biology, Institute of Development and Aging Sciences, Graduate School of Medicine, Nippon Medical School, 1-396 Kosugi-cho, Nakahara-ku, Kawasaki 211-8533, Japan; 2 Department of Applied Chemistry, National Defense Academy, 1-10-20 Hashirimizu, Yokosuka 239-8686, Japan

## Abstract

A number of alternations in mitochondrial DNA (mtDNA) have been reported in different types of cancers, and the role of mtDNA in cancer has been attracting increasing interest. In order to investigate the relationship between mtDNA alternations and chemosensitivity, we constructed cybrid (*trans*-mitochondrial hybrid) cell lines carrying a HeLa nucleus and the mtDNA of healthy individuals because of the presence of somatic alternations in the mtDNA of many cancer cells. After a treatment with 1.0 μg/mL cisplatin for 10 days, we isolated 100 cisplatin-resistant clones, 70 of which carried the shorter mtDNA OriB variant (16184–16193 poly-cytosine tract), which was located in the control region of mtDNA. Whole mtDNA sequencing of 10 clones revealed no additional alternations. Re-construction of the HeLa nucleus and mtDNA from cisplatin-resistant cells showed that cisplatin resistance was only acquired by mtDNA alternations in the control region, and not by possible alternation(s) in the nuclear genome.

A number of alternations in mitochondrial DNA (mtDNA) have been reported in different types of cancers^1^. Approximately 40~70% of cancers harbour somatic mtDNA alternations^1^, and mtDNA mutations have been shown to increase tumorigenicity^2^,^3^ and regulate tumour cell metastasis^4^. However, recent extensive mtDNA sequencing analysis of cancer biopsies using next-generation sequencer revealed that mtDNA somatic mutations have no discernible effect on cancer development, but the mutations which perturb mitochondrial functions are less likely to be maintained in cancer cells^5^.

Most anti-cancer drugs, including cisplatin (*cis*-diaminedichloroplatinum(II)) and 5-fluorouracil (5-FU), induce apoptosis^6^. It has already been established that mitochondria play important roles not only in energy production, but also in the regulation of apoptosis. We previously showed that pathogenic mtDNA mutations derived from patients with mitochondrial encephalopathy suppressed apoptosis induced by cisplatin^2^, and cells harbouring mtDNA alternations derived from human pancreatic cancer cell lines were more resistant to anti-cancer drugs than cells harbouring mtDNAs from healthy individuals^7^. Since mtDNA encodes only 13 subunits of respiratory chain complexes and all other mitochondrial proteins are encoded by genes in nuclear DNA, difficulties are associated with ensuring that the small effects attributed to mtDNA variations are not confounded by different nuclear DNA backgrounds. In order to overcome this issue, *trans*-mitochondrial hybrid cells (cybrids) with different mtDNA complements but the same nuclear DNA background were used in these studies^8^,^9^. Cybrids were generated by repopulating HeLa cells devoid of mtDNA with mtDNA derived from the enucleated cells of cisplatin-resistant clones or their parental cell. This technique allowed for the evaluation of mtDNA alternations under an identical nuclear background.

In the present study, we demonstrated that a treatment with cisplatin induced mtDNA alternations. Using cybrid technology, we showed that mtDNA alternations confer resistance to cisplatin.

## Results

### Isolation of cisplatin-resistant cells

**Figure 1:**
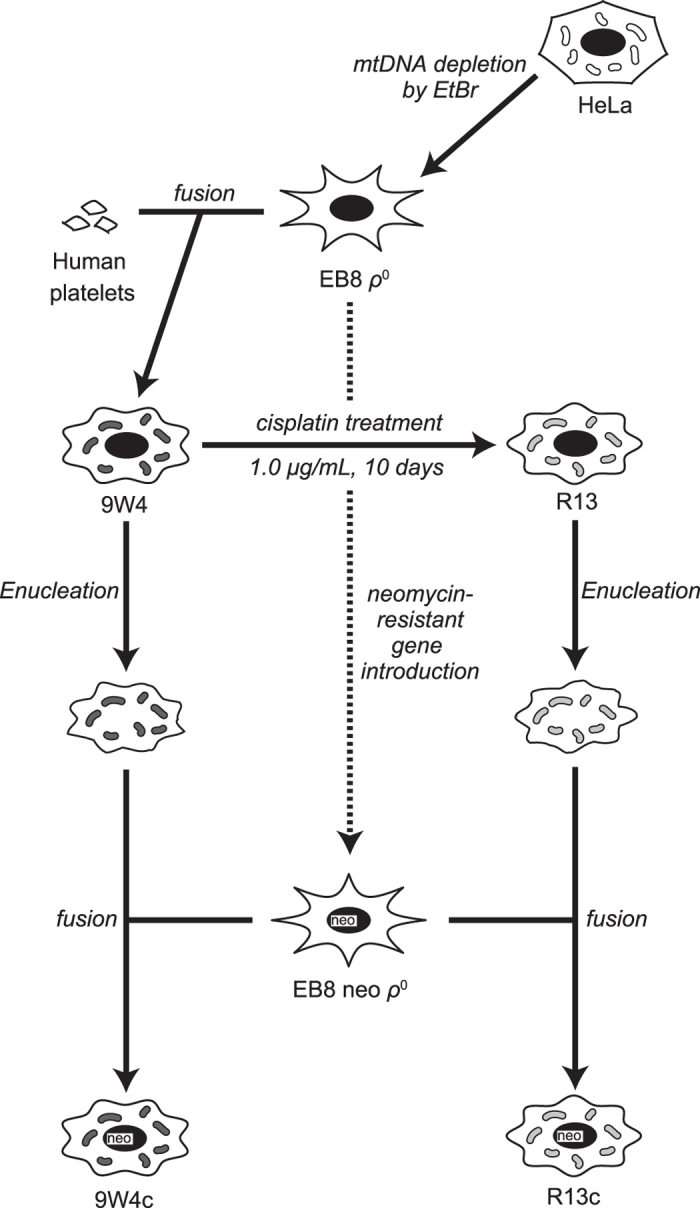
Scheme for the construction of cybrids and isolation of cisplatin-resistant cells. Since many cancer cell lines have somatic mtDNA alternations, the 9W4 cybrid, which has HeLa nuclear DNA and normal human mtDNA, was used for the cisplatin treatment. In order to evaluate the effects of mtDNA alternations caused by the cisplatin treatment, 9W4 and R13 were enucleated and fused with EB8 neo *ρ*^0^ containing a neomycin-resistant gene.

Since most cancer cells harbour somatic alternations in mtDNA and these alternations may be associated with resistance to anti-cancer drugs^10^, we used cybrid cells (A2, 8W5, 9W3, and 9W4) carrying normal mtDNA to isolate cisplatin-resistant cells. These cells (2 × 10^7^ cells each) were exposed to 1.0 μg/mL cisplatin for 10 days and continue to culture in the absence of cisplatin for additional 10 days. The resulting surviving clones were isolated as cisplatin-resistant cells (Fig. 1). We isolated 100 clones (28 clones from 8W5, 27 clones from 9W3, and 45 clones from 9W4), while no clones were obtained from A2 cybrid cells.

**Figure 2:**
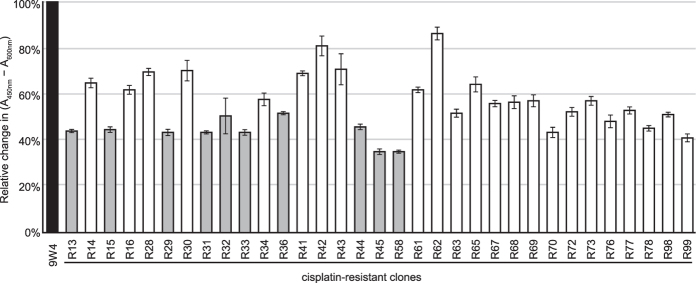
Mitochondrial dehydrogenase activities assessed by the WST-1 assay. The percentage of activity is presented relative to parental 9W4 cells. Error bars indicate S.E.M. (*n* = 3). The clones of grey columns were subjected to entire mtDNA sequencing.

The isolated clones were suspecting of having alternations in nuclear DNA as well as in mtDNA. In order to select clones with mtDNA alternations, mitochondrial dehydrogenase activities were evaluated using the WST-1 assay (Fig. 2). All assayed clones showed reductions in their mitochondrial dehydrogenase activities.

### Mitochondrial DNA sequencing to detect mtDNA alternations in cisplatin-resistant cells

**Figure 3:**
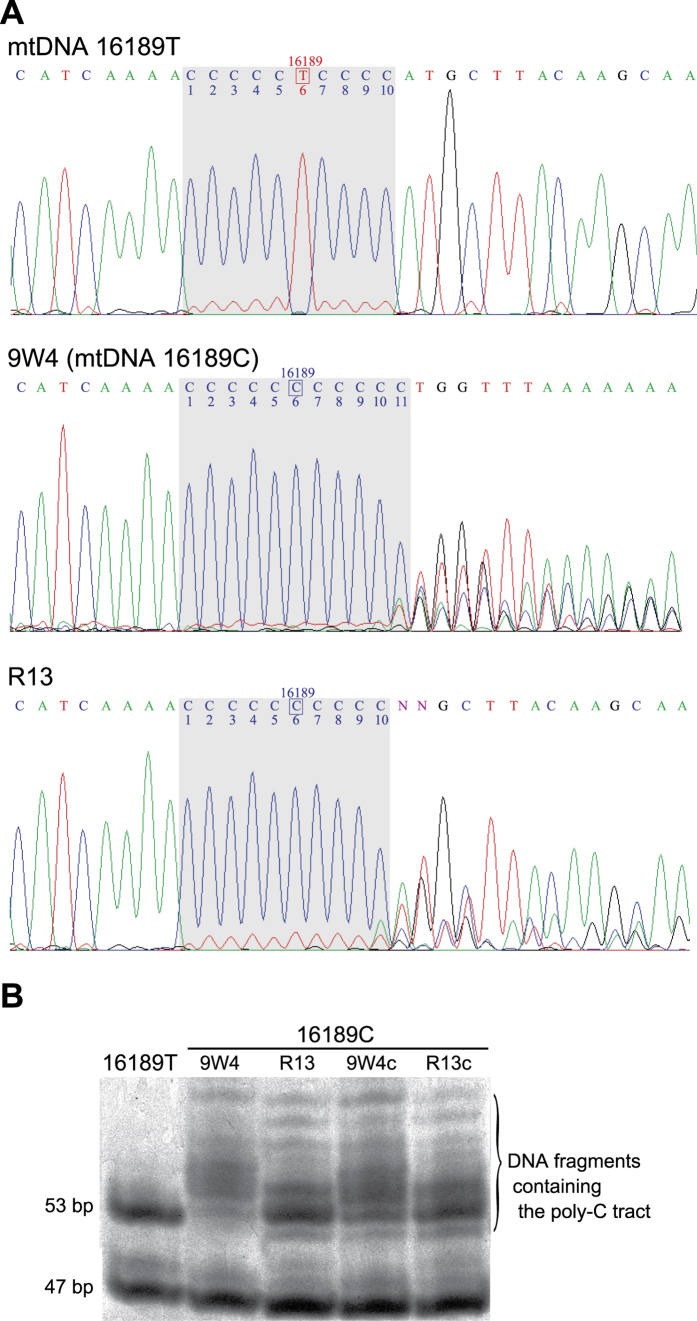
(**A**) Sequence electropherograms of mtDNA 16189 T and 16189 C. Since 9W4 cybrid has the mtDNA 16189 C variant, which causes mtDNA 16184–16193 poly-C length heteroplasmy, the electropherogram shows an unreadable sequence beyond the poly-C tract. The cisplatin-resistant R13 clone has a shorter poly-C tract than the parental 9W4 cybrid. (**B**)** A restriction fragment length polymorphism analysis of the mtDNA 16184–16193 poly-C tract.** The 53-bp DNA fragment contains the mtDNA 16184–16193 region. Full-length image is presented in Supplementary Figure S1.

We examined the entire mtDNA sequences of the parental 9W4 cybrid and 10 cisplatin-resistant clones (R13, R15, R29, R31, R32, R33, R36, R44, R45, and R58), which exhibited relatively low mitochondrial dehydrogenase activities (Fig. 2, grey columns). The 9W4 cybrid has a mtDNA T16189C variant (known as the OriB variant^11^) in the control region, which has been detected in 28.7% of Japanese individuals^12^. Furthermore, the T16189C substitution generates an uninterrupted homopolymeric cytosine tract (poly-C tract) between mtDNA nucleotide positions 16184 and 16193, and causes heteroplasmic length variations in the poly-C tract presumably due to slippage during mtDNA replication^13^. Therefore, its sequence electropherogram shows an unreadable sequence beyond the poly-C tract (Fig. 3A). The parental 9W4 cybrid showed 11 continuous cytosine peaks in the mtDNA 16184–16193 region, whereas all 10 cisplatin-resistant clones, including the R13 cybrid, only showed 10 (Fig. 3A). These 10 clones had a shorter mtDNA 16184–16193 poly-C tract than that of parental 9W4, which was confirmed by a restriction fragment length polymorphism analysis (Fig. 3B). There were no additional alternations in any of the 10 clones. Among the cybrid cell lines used for the cisplatin treatment, 8W5 and 9W3 also harbour the OriB variant. But A2, from which there were no resistant clones were obtained, does not harbour the OriB variant. Sixty out of the remaining 90 cisplatin-resistant clones were also found to have the shorter poly-C tract in a further sequencing analysis of the fragment containing the OriB variant.

### Cisplatin and 5-FU sensitivities

**Figure 4:**
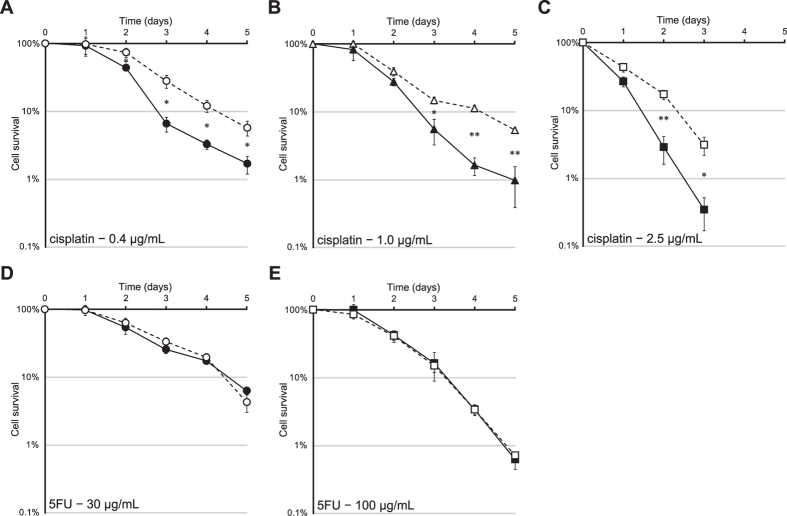
Survival of 9W4 and R13 cybrids exposed to anti-cancer drugs (cisplatin or 5-FU). (**A**) 0.4 μg/mL of cisplatin, (**B**) 1.0 μg/mL of cisplatin, (**C**) 2.5 μg/mL of cisplatin, (**D**) 30 μg/mL of 5-FU, and (**E**) 100 μg/mL of 5-FU. The cell survival fraction is given as a percentage of the respective untreated control. Closed symbols, 9W4 cybrid; open symbols, R13 cybrid. Error bars indicate S.E.M. (*n* = 3). *P < 0.05; **P < 0.01.

In order to confirm the cisplatin sensitivities of the isolated resistant clones, R13 and 9W4 cybrids were cultivated in medium containing cisplatin (Fig. 4A–C). Significant cell survival differences were observed after 2 or 3 days. However, no significant differences were noted in cell survival with the 5-FU treatment (Fig. 4D and E).

### Re-construction of cybrids

Nuclear DNA alternations have been suggested to confer cisplatin resistance as well as a shorter mtDNA poly-C tract of the OriB variant. In order to exclude this possibility, we re-transferred mtDNA from the cisplatin-resistant R13 clone to *ρ*^0^ (mtDNA-less) cells, which have nuclear DNA untreated with cisplatin, and named these cells R13c (Fig. 1). EB8 neo *ρ*^0^ cells^14^, which have a neomycin-resistant gene, were used to exclude mtDNA donor cybrids, without which it was not possible to distinguish between successfully constructed cybrids and cybrids that failed to enucleate. The nuclear DNA of parental 9W4 was also replaced in the same manner and named 9W4c (Fig. 1).

The entire mtDNA sequences of 9W4c and R13c were identical to those of their parental cells, 9W4 and R13, respectively. Additionally, their mtDNA poly-C tracts of the OriB variant exhibited similar length distributions (Fig. 3B). Therefore, the newly constructed cybrids 9W4c and R13c had identical nuclear DNA, which was not treated with cisplatin, but different length variations in their mtDNA poly-C tracts of the OriB variant.

**Figure 5:**
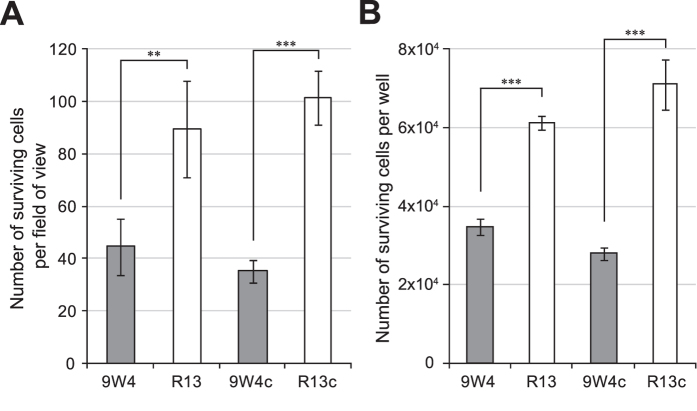
Survival assessment of re-constructed cybrids exposed to 1.0 μg/mL of cisplatin for 7 days. Cells were double-stained with Hoechst 33342 and propidium iodide. Hoechst-positive and propidium iodide-negative cells were interpreted as surviving cells. (**A**) Cells were imaged with a fluoromicroscope and counted using ImageJ. (**B**) Cells were treated with trypsin and subjected to a flow cytometric analysis. Error bars indicate S.E.M. (*n* = 3). **P < 0.01; ***P < 0.001.

In order to assess cisplatin sensitivity, 9W4c, R13c, and their parental cybrids were cultivated in the presence of 1.0 μg/mL cisplatin for 7 days. Surviving cells were counted under a fluoromicroscope after double-staining with Hoechst 33342 and propidium iodide. Newly constructed R13c cells were more resistant to cisplatin than 9W4c cells, similar to their parental cybrids (Fig. 5A), and this was confirmed by a flow cytometric analysis (Fig. 5B). These results indicate that the differences observed in cisplatin resistance between R13c and 9W4c only arose from mtDNA. Therefore, the length of the mtDNA poly-C tract of the OriB variant affects cisplatin resistance.

### Characterisation of cisplatin-resistant cybrids

**Figure 6:**
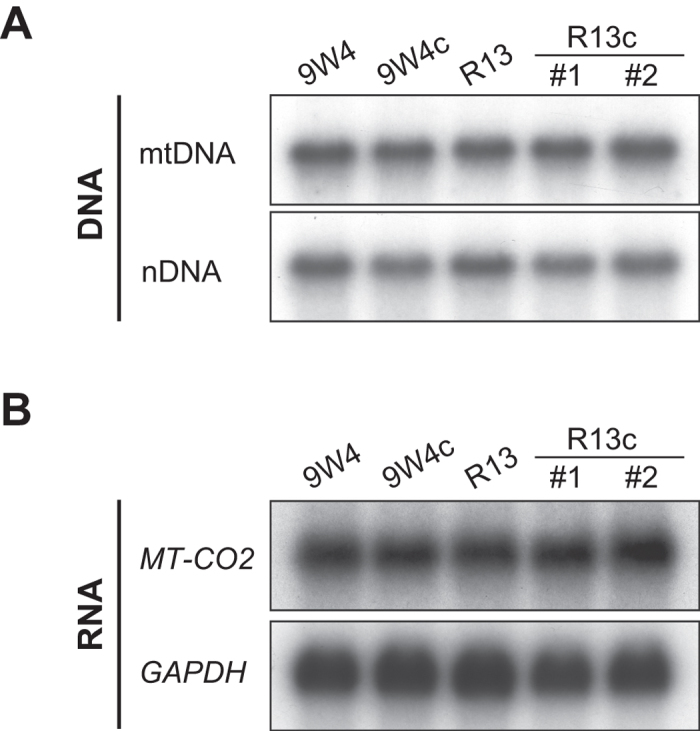
(**A**) The amount of DNA in cybrids was analysed by Southern blotting. In order to detect mtDNA, the *MT-CO2* region in mtDNA was used as a probe. Nuclear DNA (nDNA) was the loading control and the 18S ribosomal DNA region was used as the probe. Full-length blots are presented in Supplementary Figure S2. (**B**) **The expression of mtDNA and nDNA analysed by Northern blotting.** Blots of total RNA extracted from cybrids were hybridised with *MT-CO2*- and *GAPDH*-specific probes. Full-length blots are presented in Supplementary Figure S3.

Since the poly-C tract is located in the control region of mtDNA, we examined mitochondrial DNA and RNA levels in cisplatin-resistant cybrids. The amount of mtDNA was analysed by Southern blotting and no significant change was observed (Fig. 6A). Northern blotting also revealed no change in the stationary amounts of *MT-CO2* mRNA transcribed from mtDNA (Fig. 6B).

## Discussion

The results of the present study demonstrated that the length of the mtDNA poly-C tract of the OriB variant affects cisplatin resistance. The OriB variant (T16189C substitution), which is present in 10% of Europeans, 30% of Asians, 50% of Pima Indians, and >95% Polynesians^11^,^15^, generates an uninterrupted poly-C tract between mtDNA nucleotide positions 16184 and 16193. The uninterrupted poly-C tract is prone to replication slippage and creates heteroplasmic length variations within an individual^16^,^17^. 9W4, the parental cybrid cell line used in this study, harbours the 16189C variant and 16184–16193 poly-C length heteroplasmy (Fig. 3). The poly-C tract length of 9W4 was mainly longer than 10 bp (the 16189T variant) and the cisplatin treatment apparently expanded mtDNA with diminished the poly-C length. Since additional mutations were excluded by whole mtDNA sequencing and nuclear replacements, we concluded that cisplatin resistance was acquired by poly-C length alternations. This is supported by the fact that no cisplatin-resistant clones were obtained from A2 cybrid, which harbour mtDNA 16189T and does not poly-C length heteroplasmy.

The T16189C variant is now known as the OriB variant^11^ because the OriB origin of replication is located close to 16189 in the mitochondrial control region^18^. Thus, it is possible that differences in poly-C tract lengths may affect mtDNA replication and/or transcription. However, we did not detect any differences between the cisplatin-resistant R13 cybrid and its parental 9W4 cybrid (Fig. 6). The differences may be too small to be detected in the experiments conducted in the present study.

The OriB variant was reported to be associated with type 2 diabetes in Europeans and Asians^11^,^19^,^20^,^21^. However, the effects of the OriB variant may only emerge in a specific environment and/or under a specific nuclear background^22^,^23^,^24^. In the present study, we used HeLa cells as the nuclear donor. The original mitochondrial DNA of HeLa is African haplogroup L3^25^ and its nuclear DNA is presumably of an African descent. The effects of poly-C tract length variations may be masked under the nuclear background of HeLa.

Most anti-cancer drugs, including cisplatin and 5-FU, induce apoptosis^6^. Once cisplatin enters a cell with a relatively low chloride concentration, one or both of its chloride ligands are replaced by water molecules, generating a positively charged hydrated species that reacts with nucleophilic sites on intracellular macromolecules^26^. Cisplatin binds mtDNA with higher efficiency than to nuclear DNA (nDNA) presumably because positively charged species are attracted by mitochondria in response to the highly negative membrane potential^27^,^28^. Cisplatin forms covalent adducts with DNA mainly at the N-7 of guanine^29^ and preferentially forms DNA adducts at runs of consecutive guanine nucleotides (poly-G; complementary strand of poly-C)^30^. Therefore, the mtDNA poly-C tract of the OriB variant is assumed to be a primary target of cisplatin and no other mutations were found in our experiments. Cross-linked DNA with cisplatin has been suggested to trigger apoptosis^31^. 5-FU shows different mechanisms of anti-cancer action to cisplatin. It inhibits thymidylate synthase and its metabolites are incorporated into RNA and DNA^32^. Indeed, the R13 cybrid did not show resistance to 5-FU (Fig. 4).

This study showed that the length of the mtDNA poly-C tract of the OriB variant affected cisplatin resistance. It is possible that cisplatin may have directly attacked the longer poly-C tracts of mtDNA and inhibited their replication, resulting in the selection of shorter poly-C tracts. Although it was reported that the pattern of the OriB poly-C length heteroplasmy in sister cells that had been produced by replication slippage were similar^33^, cells harbouring the shorter pattern may be present at very low frequency. Such cells might be selected as the resistant cells during the treatment of cisplatin. Indeed, the resistant cells did not grow to form apparent clones.

A previous study reported that mtSSB (mitochondrial single-stranded DNA-binding protein) has lower binding affinity for the 16189C variant than the 16189T variant^20^. Since the shorter poly-C tracts selected by cisplatin were similar in length to 16189T, short poly-C tracts may have higher mtSSB affinities than long poly-C tracts and be more protective against nucleophilic attacks by cisplatin derivatives.

## Methods

### Cells and construction of cybrids

The cells used in this study were as follows: EB8 *ρ*^0^ (mtDNA-less) cells derived from human cervical cancer HeLa cells^9^; EB8 neo *ρ*^0^ cells, which were resistant to G418, were constructed by introducing a neomycin-resistant gene^14^; 8W5, 9W3, 9W4, and A2 cells were cybrid cells constructed by transferring wild-type mtDNA from three different healthy individuals into EB8 *ρ*^0^ cells^2^. Cells were cultured in Dulbecco’s modified Eagle’s medium:Nutrient Mixture F-12 (DMEM/F-12; Invitrogen, Carlsbad, CA). Uridine (50 μg/mL) was supplemented for *ρ*^0^ cells.

Cybrids were constructed as described previously^8^,^9^. Briefly, cisplatin-resistant R13 cells and their parental 9W4 cells were enucleated by exposure to cytochalasin B followed by centrifugation. The enucleated cells were fused with EB8 neo *ρ*^0^ cells with polyethylene glycol Hybri-Max (Sigma, St. Louis, MO). Cybrids were selected in medium containing G418 (Invitrogen). Binuclear cells were eliminated by a flow cytometer (Cell Lab Quanta; Beckman Coulter, Brea, CA).

### WST-1 assay

Mitochondrial dehydrogenase activity was assessed by the WST-1 assay (Cell Counting Kit; Dojindo Laboratories, Kumamoto, Japan), in which the tetrazolium salt WST-1 is converted into a coloured dye by mitochondrial dehydrogenase enzymes^34^. Briefly, 1 × 10^4^ cells in 100 μL of medium were placed in a 96-well microculture plate, and incubated at 37 °C for 2 hours. Ten microliters of WST-1 solution was then added and cells were incubated for 1 h. Optical absorbance at a test wavelength of 450 nm and reference wavelength of 600 nm was measured with a microplate reader (Immuno Mini NJ-2300; BioTec, Tokyo, Japan).

### Mitochondrial DNA sequencing and a restriction fragment length polymorphism analysis

Whole mitochondrial DNA sequencing was performed as previously described^35^. In order to assess T16189C poly-C length heteroplasmy, PCR products between mtDNA 15879 and 16545 were digested by *Afa* I and then resolved by a 20% polyacrylamide gel. The A2 cybrid, which harbours mtDNA 16189T, was used as a control of poly-C length heteroplasmy.

### Anti-cancer drug sensitivity assay

Cells were seeded on 12-well plates at a density of 3.5 × 10^4^ cells/well. After one day, medium was replaced with DMEM/F-12 containing 0.40, 1.0, and 2.5 μg/mL cisplatin (Yakult, Tokyo, Japan) or 30 and 100 μg/mL 5-FU (Sigma) plus 1.0 μM folinic acid. Cells were imaged each day and attached cells were counted using ImageJ (NIH, Bethesda, MD).

In order to assess the cisplatin sensitivity of re-constructed cybrids, cells cultivated in the presence of 1.0 μg/mL cisplatin for 7 days were double-stained with Hoechst 33342 and propidium iodide, and then imaged with a fluoromicroscope. Alternatively, double-stained cells were treated with trypsin and subjected to a flow cytometric analysis with a Cell Lab Quanta (Beckman Coulter). Hoechst-positive and propidium iodide-negative cells were interpreted as surviving cells.

### Southern and Northern blotting

Total DNA from cybrids was prepared as described previously^36^. In Southern blotting, total DNA was digested by *Pvu* II, separated by 0.8% agarose gel electrophoresis, and then transferred to a nitrocellulose membrane. [*α*-^32^P]dCTP-labelled DNA fragments corresponding to the region of *MT-CO2* (mtDNA) and 18S ribosomal DNA (nDNA) were used as probes. The oligonucleotide sequences of primers to prepare probes are shown in Supplementary Table S1.

Total RNA from cybrids was prepared using ISOGEN (Nippon Gene, Tokyo, Japan). In Northern blotting, total RNA was denatured by a heat treatment, separated by a 1.5% agarose/formaldehyde gel, and then transferred to a nitrocellulose membrane. [*α*-^32^P]dCTP-labelled DNA fragments corresponding to the region of *MT-CO2* and *GAPDH* were used as probes. The oligonucleotide sequences of primers to prepare probes are shown in Supplementary Table S1.

### Statistical Analysis

Values are presented as means ± S.E.M. (standard error of the mean). The significance of differences between means was assessed by the unpaired Student’s *t*-test; *P* values < 0.05 were considered to be significant.

## Additional Information

**How to cite this article:** Amo, T. *et al*. Cisplatin selects short forms of the mitochondrial DNA OriB variant (16184–16193 poly-cytosine tract), which confer resistance to cisplatin. *Sci. Rep.* **7**, 46240; doi: 10.1038/srep46240 (2017).

**Publisher's note:** Springer Nature remains neutral with regard to jurisdictional claims in published maps and institutional affiliations.

## Supplementary Material

Supplementary Information

## References

[b1] Kaipparettu B. A., Ma Y. & Wong L. J. Functional effects of cancer mitochondria on energy metabolism and tumorigenesis: utility of transmitochondrial cybrids. Ann. N. Y. Acad. Sci. 1201, 137–146 (2010). 20649550 10.1111/j.1749-6632.2010.05621.x

[b2] Shidara Y. . Positive contribution of pathogenic mutations in the mitochondrial genome to the promotion of cancer by prevention from apoptosis. Cancer Res. 65, 1655–1663 (2005). 15753359 10.1158/0008-5472.CAN-04-2012

[b3] Petros J. A. . mtDNA mutations increase tumorigenicity in prostate cancer. Proc. Natl. Acad. Sci. USA 102, 719–724 (2005). 15647368 10.1073/pnas.0408894102 PMC545582

[b4] Ishikawa K. . ROS-generating mitochondrial DNA mutations can regulate tumor cell metastasis. Science 320, 661–664 (2008). 18388260 10.1126/science.1156906

[b5] Ju Y. S. . Origins and functional consequences of somatic mitochondrial DNA mutations in human cancer. Elife 3 (2014). 10.7554/eLife.02935 PMC4371858 25271376

[b6] Galluzzi L. . Systems biology of cisplatin resistance: past, present and future. Cell Death Dis. 5, e1257 (2014). 24874729 10.1038/cddis.2013.428 PMC4047912

[b7] Mizutani S. . Mutations in the mitochondrial genome confer resistance of cancer cells to anticancer drugs. Cancer Sci. 100, 1680–1687 (2009). 19555391 10.1111/j.1349-7006.2009.01238.x PMC11159083

[b8] King M. P. & Attardi G. Human cells lacking mtDNA: repopulation with exogenous mitochondria by complementation. Science 246, 500–503 (1989). 2814477 10.1126/science.2814477

[b9] Hayashi J. . Introduction of disease-related mitochondrial DNA deletions into HeLa cells lacking mitochondrial DNA results in mitochondrial dysfunction. Proc. Natl. Acad. Sci. USA 88, 10614–10618 (1991). 1720544 10.1073/pnas.88.23.10614 PMC52980

[b10] Ohta S. Contribution of somatic mutations in the mitochondrial genome to the development of cancer and tolerance against anticancer drugs. Oncogene 25, 4768–4776 (2006). 16892089 10.1038/sj.onc.1209602

[b11] Ye Z. . The association of the mitochondrial DNA OriB variant (16184–16193 polycytosine tract) with type 2 diabetes in Europid populations. Diabetologia 56, 1907–1913 (2013). 23702607 10.1007/s00125-013-2945-6 PMC3737432

[b12] Kato H., Maeno Y., Ohira H., Yamada Y. & Nagao M. Molecular analysis of mitochondrial hypervariable region 1 in 394 Japanese individuals. Leg Med (Tokyo) 11, S443–445 (2009). 19254861 10.1016/j.legalmed.2009.01.069

[b13] Khogali S. S. . A common mitochondrial DNA variant associated with susceptibility to dilated cardiomyopathy in two different populations. Lancet 357, 1265–1267 (2001). 11418155 10.1016/S0140-6736(00)04422-6

[b14] Asoh S. & Ohta S. Bcl-2 completely blocks Fas-mediated apoptosis in mtDNA-depleted HeLa cells. Biochem. Biophys. Res. Commun. 237, 659–662 (1997). 9299422 10.1006/bbrc.1997.7210

[b15] Poulton J., Marchington D. R., Scott-Brown M., Phillips D. I. W. & Hagelberg E. Does a common mitochondrial DNA polymorphism underlie susceptibility to diabetes and the thrifty genotype? Trends Genet. 14, 387–389 (1998). 9820026 10.1016/s0168-9525(98)01529-7

[b16] Bendall K. E. & Sykes B. C. Length heteroplasmy in the first hypervariable segment of the human mtDNA control region. Am. J. Hum. Genet. 57, 248–256 (1995). 7668250 PMC1801530

[b17] Marchington D. R., Poulton J., Sellar A. & Holt I. J. Do sequence variants in the major non-coding region of the mitochondrial genome influence mitochondrial mutations associated with disease? Hum. Mol. Genet. 5, 473–479 (1996). 8845839 10.1093/hmg/5.4.473

[b18] Yasukawa T., Yang M. Y., Jacobs H. T. & Holt I. J. A bidirectional origin of replication maps to the major noncoding region of human mitochondrial DNA. Mol. Cell 18, 651–662 (2005). 15949440 10.1016/j.molcel.2005.05.002

[b19] Poulton J. . Type 2 diabetes is associated with a common mitochondrial variant: evidence from a population-based case-control study. Hum. Mol. Genet. 11, 1581–1583 (2002). 12045211 10.1093/hmg/11.13.1581

[b20] Park K. S. . A mitochondrial DNA variant at position 16189 is associated with type 2 diabetes mellitus in Asians. Diabetologia 51, 602–608 (2008). 18251004 10.1007/s00125-008-0933-z

[b21] Liou C. W. . Mitochondrial DNA coding and control region variants as genetic risk factors for type 2 diabetes. Diabetes 61, 2642–2651 (2012). 22891220 10.2337/db11-1369 PMC3447893

[b22] Liou C. W. . A common mitochondrial DNA variant and increased body mass index as associated factors for development of type 2 diabetes: Additive effects of genetic and environmental factors. J. Clin. Endocrinol. Metab. 92, 235–239 (2007). 17032725 10.1210/jc.2006-0653

[b23] Wallace D. C. Mitochondrial DNA variation in human radiation and disease. Cell 163, 33–38 (2015). 26406369 10.1016/j.cell.2015.08.067 PMC4743751

[b24] Latorre-Pellicer A. . Mitochondrial and nuclear DNA matching shapes metabolism and healthy ageing. Nature 535, 561–565 (2016). 27383793 10.1038/nature18618

[b25] Herrnstadt C. . A high frequency of mtDNA polymorphisms in HeLa cell sublines. Mutat. Res. 501, 19–28 (2002). 11934434 10.1016/s0027-5107(01)00304-9

[b26] Kartalou M. & Essigmann J. M. Mechanisms of resistance to cisplatin. Mutat. Res. 478, 23–43 (2001). 11406167 10.1016/s0027-5107(01)00141-5

[b27] Murata T., Hibasami H., Maekawa S., Tagawa T. & Nakashima K. Preferential binding of cisplatin to mitochondrial DNA and suppression of ATP generation in human malignant melanoma cells. Biochem. Int. 20, 949–955 (1990). 2112385

[b28] Olivero O. A., Semino C., Kassim A., Lopez-Larraza D. M. & Poirier M. C. Preferential binding of cisplatin to mitochondrial DNA of Chinese hamster ovary cells. Mutat. Res. 346, 221–230 (1995). 7753115 10.1016/0165-7992(95)90039-x

[b29] Jamieson E. R. & Lippard S. J. Structure, recognition, and processing of cisplatin-DNA adducts. Chem. Rev. 99, 2467–2498 (1999). 11749487 10.1021/cr980421n

[b30] Murray V. A survey of the sequence-specific interaction of damaging agents with DNA: emphasis on antitumor agents. Prog. Nucleic Acid Res. Mol. Biol. 63, 367–415 (1999). 10506836 10.1016/s0079-6603(08)60727-8

[b31] Yang Z. . Cisplatin preferentially binds mitochondrial DNA and voltage-dependent anion channel protein in the mitochondrial membrane of head and neck squamous cell carcinoma: possible role in apoptosis. Clin. Cancer Res. 12, 5817–5825 (2006). 17020989 10.1158/1078-0432.CCR-06-1037

[b32] Longley D. B., Harkin D. P. & Johnston P. G. 5-fluorouracil: mechanisms of action and clinical strategies. Nat. Rev. Cancer 3, 330–338 (2003). 12724731 10.1038/nrc1074

[b33] Malik S. . Evidence for the de novo regeneration of the pattern of the length heteroplasmy associated with the T16189C variant in the control (D-loop) region of mitochondrial DNA. J. Hum. Genet. 47, 122–130 (2002). 11950064 10.1007/s100380200013

[b34] Ishiyama M., Shiga M., Sasamoto K., Mizoguchi M. & He, P.-g. A new sulfonated tetrazolium salt that produces a highly water-soluble formazan dye. Chem. Pharm. Bull. 41, 1118–1122 (1993).

[b35] Chihara N. . Mitochondrial DNA alterations in colorectal cancer cell lines. J. Nippon Med. Sch. 78, 13–21 (2011). 21389643 10.1272/jnms.78.13

[b36] Amo T., Saiki S., Sawayama T., Sato S. & Hattori N. Detailed analysis of mitochondrial respiratory chain defects caused by loss of PINK1. Neurosci. Lett. 580, 37–40 (2014). 25092611 10.1016/j.neulet.2014.07.045

