# Supplementary material for: Cisplatin selects short forms of the mitochondrial DNA OriB variant (16184–16193 poly-cytosine tract), which confer resistance to cisplatin
Source: Sci Rep. 2017 Apr 10;7:46240. doi: 10.1038/srep46240 (PMC5385546; doi:10.1038/srep46240)
Supplement: Supplementary Information [file srep46240-s1.pdf]

Cisplatin selects short forms of the mitochondrial DNA OriB variant (16184-16193 poly-cytosine tract), which confer resistance to cisplatin.

Taku Amo[1,2,*], Naomi Kamimura[1], Hiromasa Asano[2], Sadamitsu Asoh[1,#], and Shigeo Ohta[1,*]

[1] Department of Biochemistry and Cell Biology, Institute of Development and Aging Sciences, Graduate School of Medicine, Nippon Medical School, 1-396 Kosugi-cho, Nakahara-ku, Kawasaki 211-8533, Japan

[2] Department of Applied Chemistry, National Defense Academy, 1-10-20 Hashirimizu, Yokosuka 239-8686, Japan

* Correspondence to Taku Amo (amo@nda.ac.jp) and Shigeo Ohta (ohta@nms.ac.jp)

[#] Present address: Hitachi High-Technologies Corporation, 882 Ichige, Hitachinaka-shi, Ibaraki, 312-8504, Japan

Supplementary Table S1 Primer sequences to prepare probes for Southern and Northern blotting

Target	Sense primer (5' - 3')	Antisense primer (5' - 3')	Application
MT-CO2	TACTTCCCCTATCATAGAAGAGCTTATCAC	GACGTCCGGGAATTGCATCTGTTTTTAAGC	Southern and Northern blotting
18S rDNA	TACCTGGTTGATCCTGCCAG	TCGGGAGTGGGTAATTTGC	Southern blotting
GAPDH	AGTCAACGGATTTGGTCGTAT	ACGATACCAAAGTTGTCATGG	Northern blotting

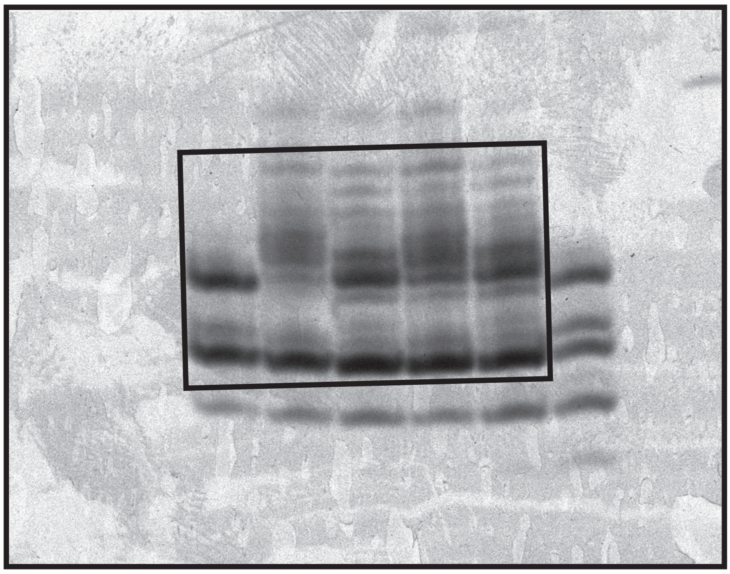

Supplementary Figure S1. Full length image of Figure 3B.

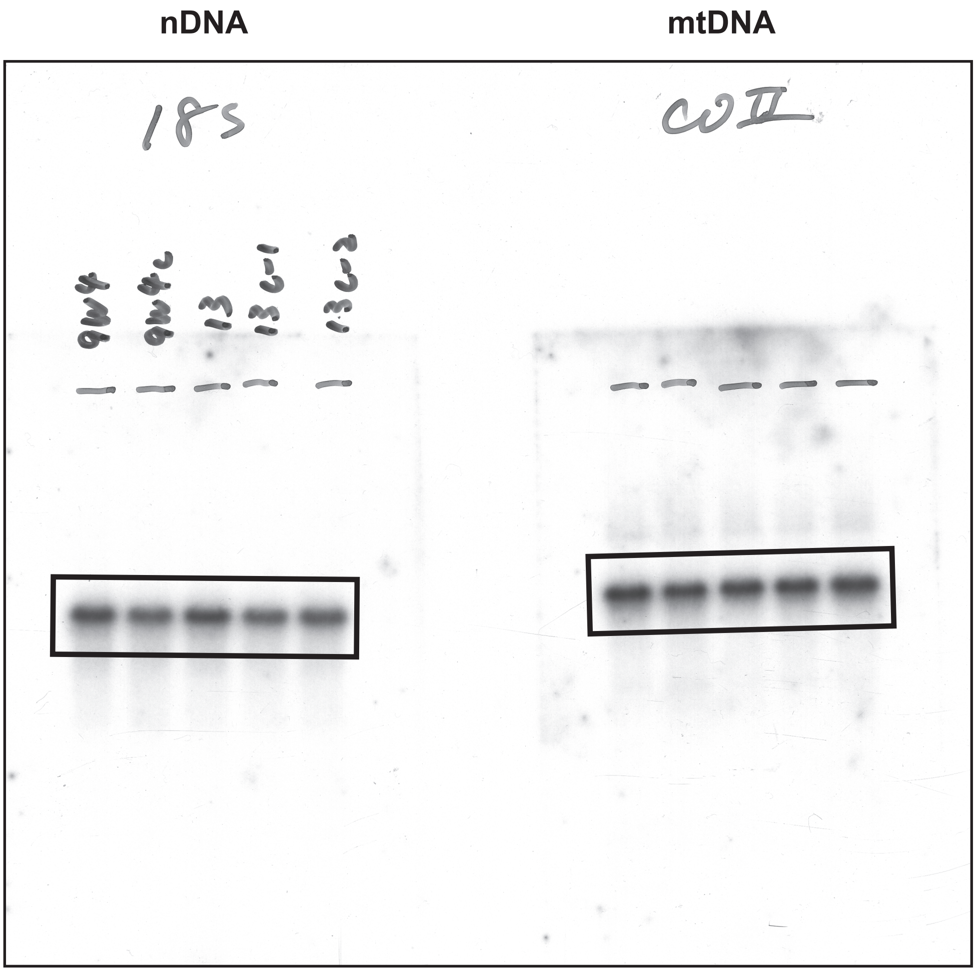

Supplementary Figure S2. Full length blots of Figure 6A.

GAPDH ***MT-CO2***

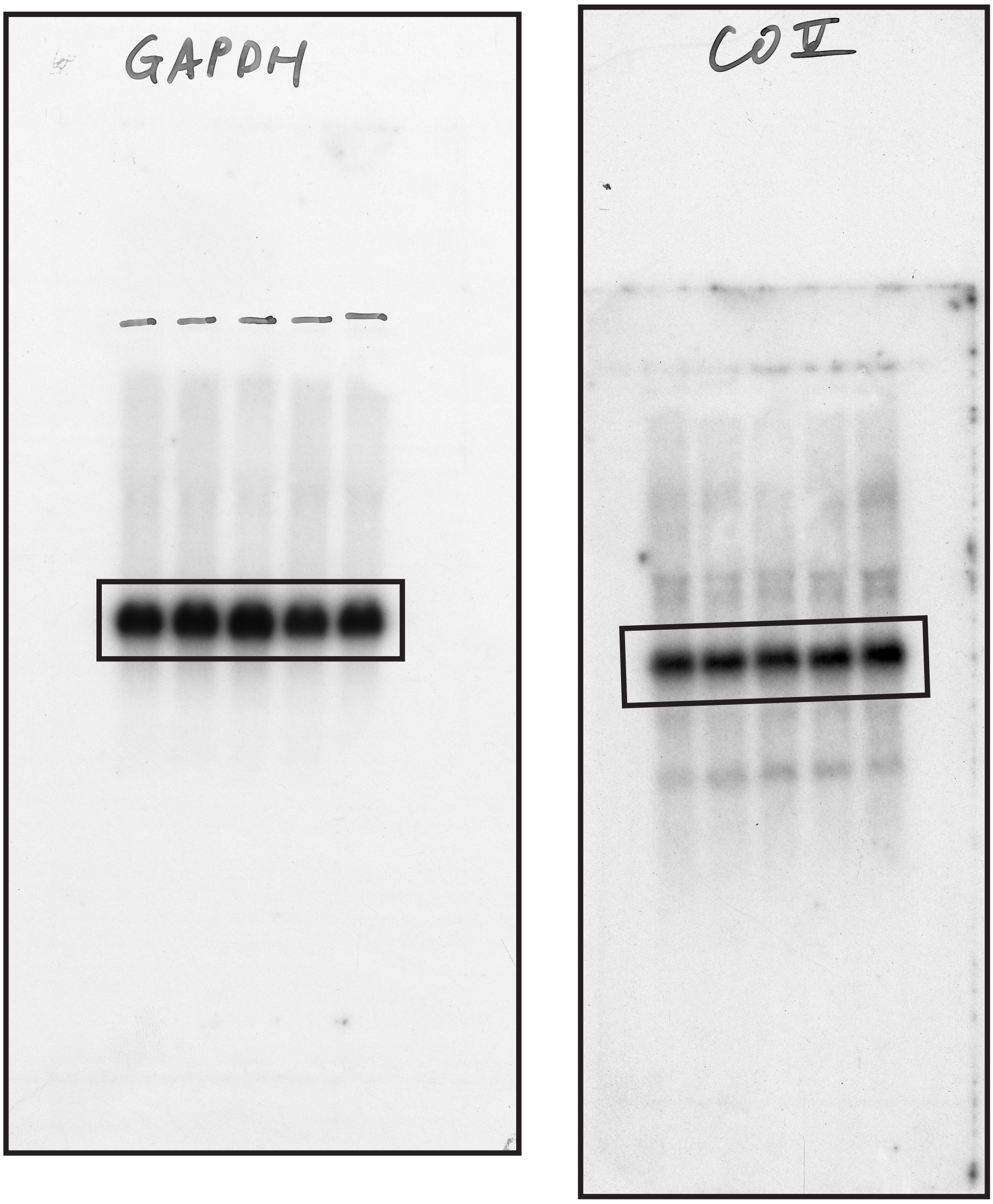

Supplementary Figure S3. Full length blots of Figure 6B.
